# Limited Usefulness of Capture Procedure and Capture Percentage for Evaluating Reproducibility in Psychological Science

**DOI:** 10.3389/fpsyg.2018.01657

**Published:** 2018-09-11

**Authors:** Yongtian Cheng, Johnson Ching-Hong Li, Xiyao Liu

**Affiliations:** ^1^Department of Psychology, University of Manitoba, Winnipeg, MB, Canada; ^2^Department of Psychology, University of Oregon, Eugene, OR, United States

**Keywords:** reproducibility, effect sizes, capture percentage, capture procedure, simulation

## Abstract

In psychological science, there is an increasing concern regarding the reproducibility of scientific findings. For instance, Replication Project: Psychology (Open Science Collaboration, 2015) found that the proportion of successful replication in psychology was 41%. This proportion was calculated based on Cumming and Maillardet (2006) widely employed *capture procedure* (CPro) and *capture percentage* (CPer). Despite the popularity of CPro and CPer, we believe that using them may lead to an incorrect conclusion of (a) successful replication when the population effect sizes in the original and replicated studies are different; and (b) unsuccessful replication when the population effect sizes in the original and replicated studies are identical but their sample sizes are different. Our simulation results show that the performances of CPro and CPer become biased, such that researchers can easily make a wrong conclusion of successful/unsuccessful replication. Implications of these findings are considered in the conclusion.

In psychological science, there is a concern regarding the replication crisis: researchers become uncertain as to whether or not a statistical finding published in the literature can be successfully replicated (Lindsay, 2015). A first approach of evaluating reproducibility lies in the *p*-value: if a *p* < 0.05, replication-study researchers consider it a successful replication of the original study, assuming the *p* < 0.05 in the original study (Appelbaum et al., 2018). However, this method is questionable because the *p*-value is not a consistent measure of an effect across replicated studies (Cumming, 2014), and the dichotomized decision (reject/do not reject a null hypothesis) results in a confusing and over-simplified view regarding the true effect in the population (Hubbard, 2011). Some journals (e.g., Basic and Applied Social Psychology) have even abandoned the use of *p*-values in their published papers.

Cumming and Maillardet (2006) suggest a second approach, where researchers evaluate the reproducibility based on an effect size (ES) and the associated CI (ESCI). That is, when the ES reported in an original study falls within the 95% CI surrounding the ES in a replicated study, then researchers can conclude that the study effect is successfully replicated. We call this *capture procedure* (CPro) in this study.

Despite researchers' efforts in providing these criteria for evaluating reproducibility, many previous projects show that the rate of successful replication is surprisingly low in psychological science. The Open Science Collaboration (2015) found that less than 50% of statistical results (e.g., *p*-value, ES) in published studies can be successfully replicated by an independent researcher. Some researchers (Baker, 2015) even call this phenomenon a *replication crisis* in the discipline.

While this low rate is alarming, we suspect that the choice of the method for evaluating reproducibility also plays a crucial role in this matter. Specifically, we believe that Cumming and Maillardet (2006) *capture percentage* (CPer), is equal the proportion of a parameter(e.g., mean or ES) of a study fall within the parameter CI of a replication study, which is equal to the proportion that CPro is successful, is only accurate when data assumptions—equal distributions of ES in the original and replicated studies (or homogeneity of original and replicated data; HORD), and homogeneity of sample sizes in the original and replicated studies (HOSS)—are assumed. The assumption of HORD has a direct effect on replication: If two datasets are coming from an identical population, then at least theoretically the results should be constant and replicate each other.

This simulation study aims to evaluate the accuracy of CPro/CPer when HORD or HOSS is violated, and to provide guidelines to researchers regarding the data conditions in which CPro/CPer is accurate. Importantly, replication researchers could evaluate whether a low reproducibility rate is due to the inappropriate use of CPro/CPer when HORD and HOSS are met or violated in practice.

## Caveat: observed Cper < 83.4% **≠** unsuccessful replication

A first large-scale replication project discussed in this paper is the Replication Project: Cancer Biology (RPCB, Mantis et al., 2017). Here, researchers hold a misconception about CI: they assume that if the replication study and the original study share an identical true distribution of scores, then the 95% CI surrounding an ES in the original study should only have a 5% likelihood that does not span the observed ESs in the replicated studies. Practically speaking, if the CPro fails in a replication attempt, RPCB researchers will view it as an important factor that the ES in the replication study is not successfully replicated. This interpretation is a good example of how researchers may misunderstand the meaning of 95% CI in concept and reproducibility research (Cumming et al., 2004): even when HORD and HOSS are met, CPer can only be 83.4 (Cumming and Maillardet, 2006).

In another project—the Replication Project: Psychology (RPP; Open Science Collaboration [OSC], 2015)—researchers found that the proportion of successful CPro is only 41% (CPer = 41%) in psychological research, which is much smaller than they expected. Most researchers would take this low rate as evidence that the majority of original studies ES cannot be successfully replicated. While RPP researchers understand that the expected CPer should be less than 95% (or failure rate = 5%) and modify the CPer standard based on the HOSS violation, they may not realize the value of CPer could still vary substantially when the condition of HORD is violated.

## Assumption A (or myth A): A high Cper or a successful CPro = HORD is met

In previous replication projects (RPP and RPCB), when the ES of the original studies falls within the CI in a replicated study, researchers will make the assumption that HORD is met, and they will conclude that the original study can be successfully replicated (CPro is successful).

If HORD is violated, which means researchers expect to observe a fail replication, the likelihood of obtaining a successful CPro in each replicated study is expected to be lower. In other words, across 1,000 replicated studies, the expected number of successful replication should be as small as possible (e.g., error rate = 5%). Hence, most researchers use CPer as a criterion for evaluating reproducibility of scientific findings. Specifically, if the CPer is smaller than 83.4%, they believe at least some studies in their project cannot be successfully replicated.

However, Cumming and Maillardet (2006) only simulated data for CPer = 83.4% when HORD and HOSS are met. When HORD is violated, no simulation, as we know, has evaluated the performance of CPer. If CPer is also reasonably high (e.g., 80%) under violated HORD, it could be questionable and debatable that a researcher concludes that the ES of a study is successfully replicated when they observe a successful CPro in their replicated study.

## Assumption B (or myth B): a low CPer or a fail CPro = HORD is violated

Sample sizes in the original and replicated studies crucially affect the value of CPer because the width of the CI depends upon the sample size in a study, and the precision of the point estimate (e.g., ES) also depends upon the sample size in a study. For instance, if the sample size is smaller in the replicated study (*n*_*r*_), then the width of the 95% CI becomes wider; at the same time, if the sample size is larger in the original study (*n*_*o*_), then the ES estimate becomes more precise. In this case, a wide CI (small *n*_*r*_) and a precise ES (large *n*_*o*_) would increase the chance of obtaining a successful CPro, and hence, the expected CPer should be higher than 83.4%. On the other hand, a narrow CI (large *n*_*r*_) and a biased ES (small *n*_*o*_) would decrease the chance of obtaining a successful CPro, and thus, the expected CPer should be smaller than 83.4%.

Fortunately, some researchers are aware of the impact of HOSS on CPer. Anderson C. J. et al. (2016) show that the mean CPer is ~ 78.5% when *n*_*r*_≠*n*_*o*_ in OSC's study, if HORD is met. Despite Anderson et al.'s findings, there is no simulation study that evaluates the behavior of CPro/CPer with different samples sizes, ESs, and distributions, so that researchers can better understand how a high (or low) CPer may not necessarily imply a successful (or unsuccessful) replication.

## Method

### Monte carlo simulation

Our purpose is to simulate how researchers typically report an ES in an original study and use CPro/CPer to examine whether the ESCI in a replicated study that spans the original ES. Given that the 2-group comparison is the most fundamental and common research scenario in behavioral research—in which researchers examine whether there is a significant difference between two groups of observation (e.g., male/female differences on cognitive ability, experimental/control group differences on reading speed, intervention/control group differences on subjective well-being, etc.)—this study focuses on simulating data for this scenario. In this case, researchers typically report Cohen's standardized mean difference *d*, i.e.,

(1) $$d=\frac{M_{1}-M_{2}}{s_{p}},$$

where (*M*_1_−*M*_2_) is the mean difference, *s*_*p*_ is the pooled standard deviation, $s_{p}\;=\;\sqrt{\left[\left(n_{1}-1\right)s_{1}^{2}+\left(n_{2}-1\right)s_{2}^{2}\right]/\left(n_{1}+n_{2}-2\right)}$, *n*_*i*_ is the sample size, and $s_{i}^{2}$ is the variance for scores in group *i* = 1, 2. When the scores deviate from normality (e.g., skewed), researchers could use the robust version of *d* (*d*_*r*_; Algina et al., 2005).

A second type of ES measures the level of association between a grouping variable and a dependent variable, which is known as point-biserial correlation (*r*_*pb*_; Ruscio, 2008). A third type of ES lies in measuring the probability-of-superiority of one group of observations over another group (*A*; Li, 2016, 2018).

For ease of presentation, we separate the simulation into the following sections. The first section evaluates the performances of CPro and CPer when the population ESs in the original and replicated studies are different (i.e., the case when HORD is violated). The purpose is to evaluate how sensitive CPro/CPer are in detecting when HORD is not met (Assumption A). The second section examines the performances of CPro/CPer when HORD is met while the HOSS is violated (Assumption B). The aim of this section is to examine how accurate CPro/CPer are in detecting HORD, when HORD is indeed met in the population, but the samples sizes are different in the original and replicated studies.

## Study 1: different population ESs in the original and replicated studies

For assumption A, we are interested in whether CPro can signal an unsuccessful replication, and whether the associated CPer becomes a small percentage because the true population ESs are different in the original and replicated studies. Ideally, CPer should be very low under this data situation. To test this assumption, we manipulated a null effect (i.e., the population standardized mean difference δ_*R*_ = 0) in the replicated study and controlled a different true δ_*o*_ (i.e., 0, 0.1, 0.2, 0.5, and 0.8) in the original study. Next, we obtained the 95% Bootstrap Bias Correlated and Accelerated Interval (BCaI; Chan and Chan, 2004) for *d*, *d*_*r*_, *r*_*pb*_, and *A* in the replicated study to form the ESCI for evaluation, given that the bootstrap procedure is widely employed by behavioral researchers. In addition to the BCaI, researchers may also construct the analytic-based CI (Cooper and Hedges, 1994) for *d* because of its simplicity and easiness in obtaining it, i.e.,

(2) $$Var_{d}\;=(\frac{n_{1}+n_{2}}{n_{1}n_{2}}+\frac{d^{2}}{2(n_{1}+n_{2}-2)})(\frac{n_{1}+n_{2}}{n_{1}+n_{2}-2}),$$

(3) $$CI_{d}\;=d\pm \sqrt{Var_{d}}*Z_{97.5\%},$$

where *n*_1_ and *n*_2_ are defined in (1), *d* is the Cohen's d, Var_d_ is the variance of d, and Z_97.5%_ is the normal cumulative distribution function (≈1.96). For each of the 5 levels of δ_*R*_ = 0 and δ_*o*_ = (0, 0.1, 0.2, 0.5, and 0.8), we evaluated 3 levels of sample sizes (25, 50, 100) and 3 levels of SD (0.5, 1, 4) in the original and replicated studies respectively, thereby producing a design with 5 × 3 × 3 = 45 conditions (for details, please see Table 1). The code is executed in RStudio (R Core Team, 2016), which is shown in Supplementary Materials.

**Table 1:** Manipulated Conditions in Simulation Study 1.

	**Original study**							**Replicated study**						
	**Group A**			**Group B**				**Group A**			**Group B**			
**Cond**	**M_o1_**	**SD_o1_**	**n_o1_**	**M_o2_**	**SD_o1_**	**n_o2_**	**δ_o_**	**M_r1_**	**SD_r1_**	**n_r1_**	**M_r2_**	**SD_r1_**	**n_r2_**	**δ_r_**
1	0	0.5	25	0	0.5	25	0	0	0.5	25	0	0.5	25	0
2	0	0.5	50	0	0.5	50	0	0	0.5	50	0	0.5	50	0
3	0	0.5	100	0	0.5	100	0	0	0.5	100	0	0.5	100	0
4	0	1	25	0	1	25	0	0	1	25	0	1	25	0
5	0	1	50	0	1	50	0	0	1	50	0	1	50	0
6	0	1	100	0	1	100	0	0	1	100	0	1	100	0
7	0	4	25	0	4	25	0	0	4	25	0	4	25	0
8	0	4	50	0	4	50	0	0	4	50	0	4	50	0
9	0	4	100	0	4	100	0	0	4	100	0	4	100	0
10	0.05	0.5	25	0	0.5	25	0.1	0	0.5	25	0	0.5	25	0
11	0.05	0.5	50	0	0.5	50	0.1	0	0.5	50	0	0.5	50	0
12	0.05	0.5	100	0	0.5	100	0.1	0	0.5	100	0	0.5	100	0
13	0.1	1	25	0	1	25	0.1	0	1	25	0	1	25	0
14	0.1	1	50	0	1	50	0.1	0	1	50	0	1	50	0
15	0.1	1	100	0	1	100	0.1	0	1	100	0	1	100	0
16	0.4	4	25	0	4	25	0.1	0	4	25	0	4	25	0
17	0.4	4	50	0	4	50	0.1	0	4	50	0	4	50	0
18	0.4	4	100	0	4	100	0.1	0	4	100	0	4	100	0
19	0.1	0.5	25	0	0.5	25	0.2	0	0.5	25	0	0.5	25	0
20	0.1	0.5	50	0	0.5	50	0.2	0	0.5	50	0	0.5	50	0
21	0.1	0.5	100	0	0.5	100	0.2	0	0.5	100	0	0.5	100	0
22	0.2	1	25	0	1	25	0.2	0	1	25	0	1	25	0
23	0.2	1	50	0	1	50	0.2	0	1	50	0	1	50	0
24	0.2	1	100	0	1	100	0.2	0	1	100	0	1	100	0
25	0.8	4	25	0	4	25	0.2	0	4	25	0	4	25	0
26	0.8	4	50	0	4	50	0.2	0	4	50	0	4	50	0
27	0.8	4	100	0	4	100	0.2	0	4	100	0	4	100	0
28	0.25	0.5	25	0	0.5	25	0.5	0	0.5	25	0	0.5	25	0
29	0.25	0.5	50	0	0.5	50	0.5	0	0.5	50	0	0.5	50	0
30	0.25	0.5	100	0	0.5	100	0.5	0	0.5	100	0	0.5	100	0
31	0.5	1	25	0	1	25	0.5	0	1	25	0	1	25	0
32	0.5	1	50	0	1	50	0.5	0	1	50	0	1	50	0
33	0.5	1	100	0	1	100	0.5	0	1	100	0	1	100	0
34	2	4	25	0	4	25	0.5	0	4	25	0	4	25	0
35	2	4	50	0	4	50	0.5	0	4	50	0	4	50	0
36	2	4	100	0	4	100	0.5	0	4	100	0	4	100	0
37	0.4	0.5	25	0	0.5	25	0.8	0	0.5	25	0	0.5	25	0
38	0.4	0.5	50	0	0.5	50	0.8	0	0.5	50	0	0.5	50	0
39	0.4	0.5	100	0	0.5	100	0.8	0	0.5	100	0	0.5	100	0
40	0.8	1	25	0	1	25	0.8	0	1	25	0	1	25	0
41	0.8	1	50	0	1	50	0.8	0	1	50	0	1	50	0
42	0.8	1	100	0	1	100	0.8	0	1	100	0	1	100	0
43	3.2	4	25	0	4	25	0.8	0	4	25	0	4	25	0
44	3.2	4	50	0	4	50	0.8	0	4	50	0	4	50	0
45	3.2	4	100	0	4	100	0.8	0	4	100	0	4	100	0

*Cond indicates a simulation condition. M_oj_, SD_oj_, and n_oj_ refer to the mean, standard deviation, and sample size, respectively for group j = 1, 2, in the original study, and M_rj_, SD_rj_, and n_rj_ refer to the mean, standard deviation, and sample size, respectively, for group j = 1, 2, in the replicated study. δ_o_ is the population standardized mean difference in the original study. δ_r_ is the standardized mean difference in the replicated study*.

In this simulation design, it is noteworthy that we simulated typical real-world conditions faced by most replication-study researchers in practice, in which ES is collected in the original study, and ESCI is collected by the replication study. (e.g., RPP). We also follow the suggestion of Unkelbach (2016) and Schweizer and Furley (2016) that the sample size of the replication study should be larger than the sample size of the original study. Step one is to find an ES observed in the original study, and step two is to find an ESCI of the replication study. We did not include the condition of using the ESCI of the original study, and the ES of the replication study because researchers typically do not report ESCI in their study. Therefore, the usage of CPro has to be based on the ES of the original study and ESCI in the replicated study. We have simulated 1,000 sample data for 1,000 observed ESs in the original study and 1,000 sample data for 1,000 observed ESCIs in the replication study. The CPer in each condition is the mean of 1,000,000 CPro, where a fail of CPer is viewed as 0, and a successful of CPer is viewed as 1.

## Results

**Figure 1:**
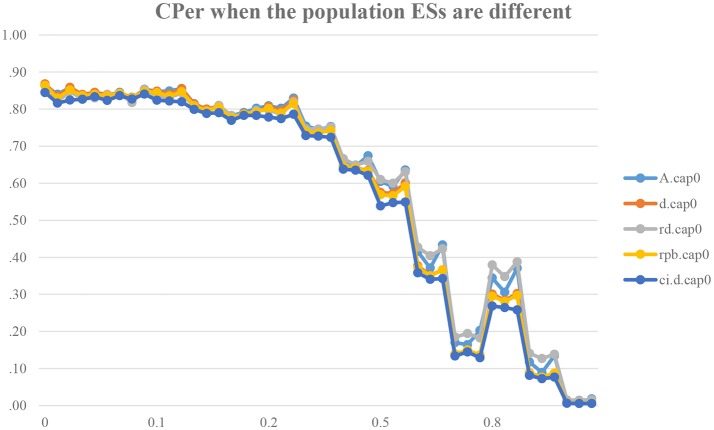
Capture Percentages across 45 conditions when the population true ESs are different. The *y*-axis shows the capture percentage. The *x*-axis shows the standardized mean difference of the original study, i.e., δ_*o*_ = (0,0.1, 0.2, 0.5, 0.8). A.cap0 is the CPer for *A*, d.cap0 is the CPer for Cohen's *d*, rd.cap0 is the CPer for robust *d* (*d*_*r*_), rpb.cap0 is the CPer for point-biserial correlation (*r*_*pb*_). The notation cap0 implies that the CPro should result in a null or *unsuccessful* (i.e., < 5%) capture procedure because the true effect sizes are different in the original and replicated studies. All these CPer methods are calculated based on the bootstrap CIs. The last term, ci.d.cap0, refers to the CPer based on the analytic CI surrounding Cohen's *d*.

We expect that CPer would ideally become low (e.g., .05) when there is a difference between δ_*o*_ and δ_*R*_ (i.e., δ = δ_*o*_−δ_*R*_). However, as shown in Figure 1, CPer is found to be around 80% when δ = 0.1, CPer ≈ 75% when δ = 0.2, CPer ≈ 45% when δ = 0.5, and CPer ≈ 25% when δ = 0.8. Taking a scenario that a replication-study researcher would like to use CPro for testing whether a study effect can be successfully replicated: when δ = 0.1, this researcher has 80% likelihood (or 4 out of 5) that the ES in the original study falls within the 95% ESCI in the replicated study. However, there is a difference between the true ESs in the original and replicated studies. When data generates from δ = 0.1 (instead of δ = 0), the researcher, in theory, should conclude that the ES in the replicated study cannot replicate the ES in the original study. However, in practice, researchers are likely to conclude that the ES in the original study can be successfully replicated because of a relatively large CPer (i.e., 80%) in the long run. This raises a concern about the adequate use of CPer in judging and concluding whether or not δ = 0 is tenable, especially when δ is slightly larger than 0. Even when δ = 0.2, which is equal to a change from a zero to small ES (*d* = 0 is interpreted as a null effect; *d* = 0.2 is interpreted as a small ES; Cohen, 1988), the expected CPer is around 75%, meaning that replication-study researchers have a 75% likelihood of (inappropriately) concluding that a study effect can be successfully replicated. However, the true ES is small (δ_*o*_ = 0.2) in the original study and true effect is zero (δ_*R*_ = 0.2) in the replicated study. We also found that there is a difference between five different ES and ESCI measurement methods, but there is no single method that is robust to the violation of Assumption A.

## Study 2: different sample sizes in the original and replicated studies

In this simulation, we evaluate whether CPro/CPer can appropriately signal a successful replication when HORD is met (e.g., δ = 0), but the HOSS is violated. We expect that CPer should have 83.4% likelihood leading to a conclusion that an ES in the replicated study can be successfully replicated (i.e., δ = 0). On the other hand, if CPer becomes much smaller than 83.4%, there is a serious concern regarding the appropriate use of CPro/CPer in replication research.

To determine this, we manipulated 5 levels of δ_*o*_ = δ_*r*_ = (0, 0.1, 0.2, 0.5, and 0.8), 3 levels of sample sizes in the original study (*n*_*o*1_, *n*_*o*2_) = (25, 25), (50, 50), and (100, 100), 1 level of sample size in the replication study (*n*_*r*1_, *n*_*r*2_) = (100, 100), and 3 levels of SDs in the original and replicated studies (0.25, 1, 4), thereby producing a design with 5 × 3 × 1 × 3 = 45 conditions (for details, please see Table 2). The code is shown in the Supplementary Materials. The inclusion of ES and ESCI measurement methods, and the calculation of CPer remains the same as in the first simulation study.

**Table 2:** Manipulated Conditions in Simulation Study 2.

	**Original study**							**Replicated study**						
	**Group A**			**Group B**				**Group A**			**Group B**			
**Cond**	**M_o1_**	**SD_o1_**	**n_o1_**	**M_o2_**	**SD_o1_**	**n_o2_**	**δ_o_**	**M_r1_**	**SD_r1_**	**n_r1_**	**M_r2_**	**SD_r1_**	**n_r2_**	**δ_r_**
1	0	0.5	25	0	0.5	25	0	0	0.5	100	0	0.5	100	0
2	0	0.5	50	0	0.5	50	0	0	0.5	100	0	0.5	100	0
3	0	0.5	100	0	0.5	100	0	0	0.5	100	0	0.5	100	0
4	0	1	25	0	1	25	0	0	1	100	0	1	100	0
5	0	1	50	0	1	50	0	0	1	100	0	1	100	0
6	0	1	100	0	1	100	0	0	1	100	0	1	100	0
7	0	4	25	0	4	25	0	0	4	100	0	4	100	0
8	0	4	50	0	4	50	0	0	4	100	0	4	100	0
9	0	4	100	0	4	100	0	0	4	100	0	4	100	0
10	0.05	0.5	25	0	0.5	25	0.1	0.05	0.5	100	0	0.5	100	0.1
11	0.05	0.5	50	0	0.5	50	0.1	0.05	0.5	100	0	0.5	100	0.1
12	0.05	0.5	100	0	0.5	100	0.1	0.05	0.5	100	0	0.5	100	0.1
13	0.1	1	25	0	1	25	0.1	0.1	1	100	0	1	100	0.1
14	0.1	1	50	0	1	50	0.1	0.1	1	100	0	1	100	0.1
15	0.1	1	100	0	1	100	0.1	0.1	1	100	0	1	100	0.1
16	0.4	4	25	0	4	25	0.1	0.4	4	100	0	4	100	0.1
17	0.4	4	50	0	4	50	0.1	0.4	4	100	0	4	100	0.1
18	0.4	4	100	0	4	100	0.1	0.4	4	100	0	4	100	0.1
19	0.1	0.5	25	0	0.5	25	0.2	0.1	0.5	100	0	0.5	100	0.2
20	0.1	0.5	50	0	0.5	50	0.2	0.1	0.5	100	0	0.5	100	0.2
21	0.1	0.5	100	0	0.5	100	0.2	0.1	0.5	100	0	0.5	100	0.2
22	0.2	1	25	0	1	25	0.2	0.2	1	100	0	1	100	0.2
23	0.2	1	50	0	1	50	0.2	0.2	1	100	0	1	100	0.2
24	0.2	1	100	0	1	100	0.2	0.2	1	100	0	1	100	0.2
25	0.8	4	25	0	4	25	0.2	0.8	4	100	0	4	100	0.2
26	0.8	4	50	0	4	50	0.2	0.8	4	100	0	4	100	0.2
27	0.8	4	100	0	4	100	0.2	0.8	4	100	0	4	100	0.2
28	0.25	0.5	25	0	0.5	25	0.5	0.25	0.5	100	0	0.5	100	0.5
29	0.25	0.5	50	0	0.5	50	0.5	0.25	0.5	100	0	0.5	100	0.5
30	0.25	0.5	100	0	0.5	100	0.5	0.25	0.5	100	0	0.5	100	0.5
31	0.5	1	25	0	1	25	0.5	0.5	1	100	0	1	100	0.5
32	0.5	1	50	0	1	50	0.5	0.5	1	100	0	1	100	0.5
33	0.5	1	100	0	1	100	0.5	0.5	1	100	0	1	100	0.5
34	2	4	25	0	4	25	0.5	2	4	100	0	4	100	0.5
35	2	4	50	0	4	50	0.5	2	4	100	0	4	100	0.5
36	2	4	100	0	4	100	0.5	2	4	100	0	4	100	0.5
37	0.4	0.5	25	0	0.5	25	0.8	0.4	0.5	100	0	0.5	100	0.8
38	0.4	0.5	50	0	0.5	50	0.8	0.4	0.5	100	0	0.5	100	0.8
39	0.4	0.5	100	0	0.5	100	0.8	0.4	0.5	100	0	0.5	100	0.8
40	0.8	1	25	0	1	25	0.8	0.8	1	100	0	1	100	0.8
41	0.8	1	50	0	1	50	0.8	0.8	1	100	0	1	100	0.8
42	0.8	1	100	0	1	100	0.8	0.8	1	100	0	1	100	0.8
43	3.2	4	25	0	4	25	0.8	3.2	4	100	0	4	100	0.8
44	3.2	4	50	0	4	50	0.8	3.2	4	100	0	4	100	0.8
45	3.2	4	100	0	4	100	0.8	3.2	4	100	0	4	100	0.8

*Cond indicates a simulation condition. M_oj_, SD_oj_, and n_oj_ refer to the mean, standard deviation, and sample size, respectively for group j = 1, 2, in the original study, and M_rj_, SD_rj_, and n_rj_ refer to the mean, standard deviation, and sample size, respectively, for group j = 1, 2, in the replicated study. δ_o_ is the population standardized mean difference in the original study. δ_r_ is the standardized mean difference in the replicated study*.

## Results

**Figure 2:**
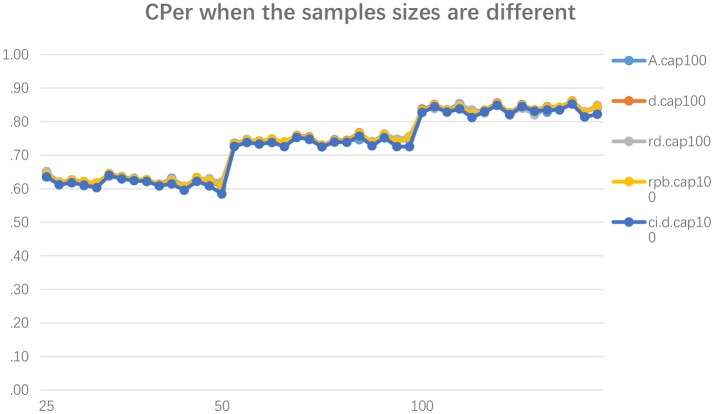
Capture Percentages across 45 conditions when the sample sizes in the original and replicated studies are different. The *y*-axis shows the capture percentage. The *x*-axis is the sample sizes for the original study. A.cap0 is the CPer for *A*, d.cap100 is the CPer for Cohen's *d*, rd.cap100 is the CPer for robust *d* (*d*_*r*_), rpb.cap100 is the CPer for point-biserial correlation (*r*_*pb*_). The notation cap100 implies that the capture procedure should result in a successful (i.e., about 83.4%) capture procedure because the true effect sizes are the same in the original and replicated studies. All these CPer methods are calculated based on the bootstrap CIs. The last term, ci.d.cap0, refers to the CPer based on the analytic CI surrounding Cohen's *d*.

Based on the results in Figure 2, in general, when the sample size of the replication study is twice as large as the original study, and the population ES of the original study and replication study are identical, the CPer is about 73%. When the sample size of the replication study is four times larger than the original study in this condition, the CPer is about 60%. Both are significantly different from the CPer when the sample sizes of the original study and replication study are equal. There is no noticeable difference found between these conditions in each sample size's difference condition or ES and ESCI measurement method.

In sum, the use of CPro and CPer as a criterion for judging whether a study effect can be successfully replicated is highly questionable, given that CPer is significantly influenced by the sample size difference between original studies and replication studies. If researchers want to increase the sample size in the replication study, then CPer should not be used to test whether the ES of the original study is replicated by the replication study.

## Discussion

This study examines whether the use of CPro/CPer is a legitimate procedure in concluding that an ES in the replicated study is a successful replication of the ES in the original study, when HORD or HOSS is met or violated. The results show that CPer can easily and inappropriately become very close to the criterion of 83.4% under violated HORD (e.g., as high as 82%; Figure 1), and CPer can easily and inappropriately become smaller than the criterion of 83.4% under violated HOSS (e.g., as low as 61% in Figure 2). Consider this example: if a researcher finds that an observed CPer is 70%, then the researcher often cannot make a correct decision (δ = 0 or δ≠0) because this value could be possible under either condition.

### Is CPro/CPer always a consistent measure of reproducibility?

We believe that the use of CPro/CPer is debatable and questionable. As an analogy, when researchers use frequentist statistical reasoning to make a statistical inference (reject/accept a null hypothesis), they should first assume that the null hypothesis (*H*_0_) is correct (e.g., lack of effect), and see how a sampled ES behaves when *H*_0_ is true. When a sampled ES deviates substantially from the expected distribution given *H*_0_ (i.e., |*sampled ES*| > critical ES), then the researchers should reject *H*_0_. The condition of *H*_0_ is crucial because researchers should adopt a conservative approach and assume a zero effect; unless they observe an ES deviated from a zero effect, they cannot conclude that a significant ES (their target outcome) exists in their research.

For the case of CPro/CPer, a researcher's typical target outcome is *successful replication*. Theoretically, the pre-requisite condition should be the opposite (unequal distributions in the original and replicated studies). However, CPro/CPer are operating differently—researchers first assume *equal distributions*, and next, they observe whether the ES in the original study falls within the 95% ESCI in the replicated study. Undoubtedly, when the condition of “*H*_0_: equal distributions” is true, then there is a good chance for researchers to observe the consequence that the ES in the original study falls within the 95% ESCI, i.e.,

(4) $$\begin{array}{l} P\left(\text{ES falling within CI}|{\text{H}}_{0}=\text{equal distributions}\right)\\ \;=P\left(\text{ES falling within CI}|{\text{H}}_{0}\text{ is true}\right)\\ \;=P\left(\text{successful CPro}|{\text{H}}_{0}\text{ is true}\right)\\ \;=CPer \end{array}$$

Of the 1,000 replicated studies (with the same sample size) sampled from the same underlying distribution, Cumming and Maillardet (2006) showed that there should be around 834 studies containing a successful CPro. However, there are two issues regarding this interpretation. First, evaluating whether an ES in the original study falling within the ESCI is a natural consequence of (but not a decision-making process for concluding) *H*_0_ = *equal distributions*, and this evaluation does not provide any information regarding how likely *H*_0_ is false (i.e., unequal distributions). An analogy of CPer is similar to Power *P*(|*sampled ES*| > *critical ES* |*H*_1_ *is true*), in which Power only informs researchers how likely it is that they observe a significant result given that *H*_1_ *is true*. Hence, using CPer to evaluate whether reproducibility is true in a given population may evoke a logical fallacy. Logically, *P*(*ES falling within CI*|*H*_0_ = *unequal distributions*) should be the parameter that researchers are seeking.

Second, (4) shows that using CPer = 83.4% as a criterion for successful replication is overly simplified. The expected value of 83.4% is true if and only if HORD and HOSS are met. In practice, it is likely that the original and replicated study samples originate from (slightly) different distributions, and these studies have different sample sizes. Our simulation results show that CPer could become a large value when δ is small (e.g., 0.1, or 0.2) with *n*_*r*_ = *n*_*o*_, but it could become a small value when δ = 0 with *n*_*r*_≠*n*_*o*_, thus suggesting that CPer is not a consistent measure to evaluate reproducibility.

### Implications of the findings

#### Theoretical researchers

We encourage theoretical researchers to develop alternative measures to CPro/CPer for evaluating the replicability of research findings. For example, we suggest that researchers consider equivalence testing (Goertzen and Cribbie, 2010; Anderson S. F. et al., 2016) that specifies an acceptable range of δ that is considered a successful replication. Instead of using δ = 0 as an absolute criterion, researchers could specify a reasonable range of acceptance, e.g.,[δ] ≤ 0.2. This means that if the true ES in the replicated study does not deviate more than .2 units relative to the true ES in the original study, then the researcher should regard the result as a reasonable replication. Another alternative approach to solving the issue with *P*(*successful CPro*|*H*_0_ *is true*) is the use of Bayesian statistics, which could reverse the marginal probabilities in (4) to become a more conceptually correct evaluation of reproducibility.

#### Applied researchers

In the meantime, without other alternatives, applied researchers should pay attention to the conceptual meaning of CPer = *P*(*successful CPro*|*H*_0_ *is true*). That is, applied researchers could obtain a CPer slightly smaller but still close to the criterion of 83.4%, when δ is small (e.g., 0.1, or 0.2) with the same sample sizes in the original and replicated studies. At the same time, researchers could obtain a CPer much smaller than the criterion of 83.4%, when δ = 0 with different sample sizes in the original and replicated studies. In short, we encourage applied researchers to avoid using CPro/CPer as the sole criterion in evaluating reproducibility. Finally, because that both CPro and CPer are problematic as shown in the current simulation study, but CPer results have been widely employed by replication-study researchers (e.g., RPCB, Valentine et al., 2011), we encourage researchers to find a more appropriate interpretation and better explanation for these results. For example, in replication studies of Currency Priming (Caruso et al., 2013) and Flag Priming (Carter et al., 2011; Study 2) in the Many Labs project (Klein et al., 2014) researchers have found that most of the mean or median ESs of there replication studies are at or even below the lower bound of the 95% ESCIs in the original studies. These results are highly incompatible with the current model of common practice in which original studies and replication studies always share an identical distribution prior to data collection. To better interpret these results, researchers should conduct more research in order to find out whether this pattern of result is due to *the criterion* (i.e., CPro and CPer) they used for evaluating reproducibility, or whether there is a real *replication crisis* in these replication studies.

## Author contributions

YC is responsible for the generation of the research ideas, review of the existing literature, design of the simulation study, and report and interpretation of the results. JL is the academic advisor of YC, and he provides advice in developing the purposes and writing the contents of the study, evaluation of the simulation design, and integration of the simulation results with the theory about reproducibility in psychological science. XL is responsible for providing advice to the writing and contents of the studies related to replication crisis and issues regarding reproducibility.

### Conflict of interest statement

The authors declare that the research was conducted in the absence of any commercial or financial relationships that could be construed as a potential conflict of interest.
