# Supplementary material for: Limited Usefulness of Capture Procedure and Capture Percentage for Evaluating Reproducibility in Psychological Science
Source: Front Psychol. 2018 Sep 11;9:1657. doi: 10.3389/fpsyg.2018.01657 (PMC6141826; doi:10.3389/fpsyg.2018.01657)
Supplement: Supplementary file 1 [file Data_Sheet_1.DOCX]

**Supplementary Materials – The R Rode Used in the Simulation Study**

**Study 1**

# the whole code is written by Yongtian Cheng, for the capture percentage project#

#there is some unused function.

##all the 2(alternative/null capture percentage)*5 are very similar, the difference will be noticed.#

install.packages("boot", dependencies = TRUE)

install.packages("lsr", dependencies = TRUE)

install.packages("doParallel", dependencies = TRUE)

install.packages("MBESS", dependencies = TRUE)

install.packages("psych", dependencies = TRUE)

library(MBESS)

library(boot)

library(lsr)

library(psych)

library(doParallel)

clus.h=4

boottime=1000

simutime=1000

cll <- makeCluster(clus.h)

registerDoParallel(cll)

set.seed(201701)

#get significance of P-value

get.p=function(a,b){

sig.test=t.test(a,b,paired = FALSE,var.equal = FALSE)$p.value

if(sig.test<0.05){sig.use=1}

if(sig.test>=0.05){sig.use=0}

return(sig.use)

}

#calculate ES of A,D,RD,RPB#(some other redudent code are orginal degsin.)

#we used to think do a transformation of z-value would be interesting, however, it

#does not make sence.

get.amethod=function(dat){

Y1 <- dat[which(dat[,1] ==1, arr.ind = T),2]

Y2 <- dat[which(dat[,1] ==2, arr.ind = T),2]

part1=length(Y1)

part2=length(Y2)

countA=0

ut1=1

while(ut1<=part1){

ut2=1

while(ut2<=part2){

if(Y1[ut1]>Y2[ut2]){

countA=countA+1

}

if(Y1[ut1]==Y2[ut2]){

countA=countA+0.5

}

ut2=ut2+1

}

ut1=ut1+1

}

amethod=countA/(part1*part2)

return(amethod)

}

get.d=function(dat){

likeY1 <- dat[which(dat[,1] ==1, arr.ind = T),2]

likeY2 <- dat[which(dat[,1] ==2, arr.ind = T),2]

p1=(length(likeY1))/(length(likeY1)+length(likeY2))

p2=(length(likeY2))/(length(likeY1)+length(likeY2))

cohend=(mean(likeY1)-mean(likeY2))/((p1*var(likeY1)+p2*var(likeY2))^0.5)

return(cohend)

}

get.rd=function(dat){

likeY1 <- dat[which(dat[,1] ==1, arr.ind = T),2]

likeY2 <- dat[which(dat[,1] ==2, arr.ind = T),2]

part1=length(likeY1)

part2=length(likeY2)

robustv=(winsor.var(likeY1,trim=0.2)*(part1-1)+winsor.var(likeY2,trim=0.2)*(part2-1))/(part1+part2-2)

cohendr=0.642*((mean(likeY1,trim=0.2)-mean(likeY2,trim=0.2))/((robustv)^0.5))

return(cohendr)

}

get.rpb=function(dat){

likeY1 <- dat[which(dat[,1] ==1, arr.ind = T),2]

likeY2 <- dat[which(dat[,1] ==2, arr.ind = T),2]

p1=(length(likeY1))/(length(likeY1)+length(likeY2))

p2=(length(likeY2))/(length(likeY1)+length(likeY2))

rpb=(mean(likeY1)-mean(likeY2))/((p1*var(likeY1)+p2*var(likeY2))/(p1*p2)+(mean(likeY1)-mean(likeY2))^2)^0.5

return(rpb)}

get.eta=function(dat){

likeY1 <- dat[which(dat[,1] ==1, arr.ind = T),2]

likeY2 <- dat[which(dat[,1] ==2, arr.ind = T),2]

likeall=c(likeY1,likeY2)

ssbetween=(mean(likeall)-mean(likeY1))^2*length(likeY1)+(mean(likeall)-mean(likeY2))^2*length(likeY2)

sstotal=sum((likeY1-mean(likeall))^2)+sum((likeY2-mean(likeall))^2)

eta=ssbetween/sstotal

return(eta)}

get.r.z=function(dat){

cor.u=cor(dat[,1],dat[,2])

z.u=(log(1+cor.u)-log(1-cor.u))/2

return(z.u)

}

#bootstarp function for different ESs

frame.boot=function(Y1,Y2){

n1 <- length(Y1)

n2 <- length(Y2)

n <- n1+n2

dat <- array(0, dim=c(n,2))

dat[1:n1,1] <- 1

dat[(n1+1):n,1] <- 2

dat[1:n1,2] <- Y1

dat[(n1+1):n,2] <- Y2

return(dat)

}

f.a <- function(d, i){

d2 <- d[i,]

return(get.amethod(d2))

}

f.d <- function(d, i){

d2 <- d[i,]

return(get.d(d2))

}

f.rd <- function(d, i){

d2 <- d[i,]

return(get.rd(d2))

}

f.rpb <- function(d, i){

d2 <- d[i,]

return(get.rpb(d2))

}

f.eta <- function(d, i){

d2 <- d[i,]

return(get.eta(d2))

}

f.r.z <- function(d, i){

d2 <- d[i,]

return(get.r.z(d2))

}

A.bca.ci=function(Y1,Y2){

dat=frame.boot(Y1,Y2)

boot.data <- boot(dat, f.a, R=boottime, parallel = c("snow"), cl=clus.h)

save.all=boot.ci(boot.data, type = c("bca"))

save.min=save.all$"bca"[4]

save.max=save.all$"bca"[5]

return(c(save.min,save.max))

}

d.bca.ci=function(Y1,Y2){

dat=frame.boot(Y1,Y2)

boot.data <- boot(dat, f.d, R=boottime, parallel = c("snow"), cl=clus.h)

save.all=boot.ci(boot.data, type = c("bca"))

save.min=save.all$"bca"[4]

save.max=save.all$"bca"[5]

return(c(save.min,save.max))

}

rd.bca.ci=function(Y1,Y2){

dat=frame.boot(Y1,Y2)

boot.data <- boot(dat, f.rd, R=boottime, parallel = c("snow"), cl=clus.h)

save.all=boot.ci(boot.data, type = c("bca"))

save.min=save.all$"bca"[4]

save.max=save.all$"bca"[5]

return(c(save.min,save.max))

}

rpb.bca.ci=function(Y1,Y2){

dat=frame.boot(Y1,Y2)

boot.data <- boot(dat, f.rpb, R=boottime, parallel = c("snow"), cl=clus.h)

save.all=boot.ci(boot.data, type = c("bca"))

save.min=save.all$"bca"[4]

save.max=save.all$"bca"[5]

return(c(save.min,save.max))

}

eta.bca.ci=function(Y1,Y2){

dat=frame.boot(Y1,Y2)

boot.data <- boot(dat, f.eta, R=boottime, parallel = c("snow"), cl=clus.h)

save.all=boot.ci(boot.data, type = c("bca"))

save.min=save.all$"bca"[4]

save.max=save.all$"bca"[5]

return(c(save.min,save.max))

}

r.z.bca.ci=function(Y1,Y2){

dat=frame.boot(Y1,Y2)

boot.data <- boot(dat, f.r.z, R=boottime, parallel = c("snow"), cl=clus.h)

save.all=boot.ci(boot.data, type = c("bca"))

save.min=save.all$"bca"[4]

save.max=save.all$"bca"[5]

return(c(save.min,save.max))

}

check.include=function(test,min,max){

ans=0

if(test>max){ans=1}

if(test<min){ans=1}

return(ans)}

check.cap=function(resv,minv,maxv){

ans.u=0

resv.n=1

while(resv.n<=length(resv)){

resu=resv[resv.n]

min.v=1

while(min.v<=length(minv)){

minu=minv[min.v]

maxu=maxv[min.v]

ans.u=ans.u+check.include(resu,minu,maxu)

min.v=min.v+1}

resv.n=resv.n+1}

return(ans.u)}

######################

vsample1=c(25,50,100)

vsample2=c(1)

vsd1=c(0.5,1,4)

vsd2=c(1)

vd=c(0,0.1,0.2,0.5,0.8)

samplecount1=1

samplecount2=1

sdcount1=1

sdcount2=1

dcount=1

times=1

saveall=1

condition=1

while(samplecount1<=length(vsample1)){

while(samplecount2<=length(vsample2)){

while(sdcount1<=length(vsd1)){

while(sdcount2<=length(vsd2)){

while(dcount<=length(vd)){

vsample2=vsample1[samplecount1]

p1=(vsample1[samplecount1])/(vsample1[samplecount1]+vsample2[samplecount2])

p2=(vsample2[samplecount2])/(vsample1[samplecount1]+vsample2[samplecount2])

pooledsd=(vsd1[sdcount1]^2*p1+vsd2[sdcount2]^2*p2)^0.5

mean1=(vd[dcount])*pooledsd

save.a=1

save.d=1

save.rd=1

save.rpb=1

save.eta=1

save.r.z=1

save.a.bca.min=1

save.a.bca.max=1

save.d.bca.min=1

save.d.bca.max=1

save.rd.bca.min=1

save.rd.bca.max=1

save.rpb.bca.min=1

save.rpb.bca.max=1

save.d.ci.min=1

save.d.ci.max=1

save.r.z.bca.min=1

save.r.z.bca.max=1

save.r.z.ci.min=1

save.r.z.ci.max=1

save.a.length=1

save.d.length=1

save.rpb.length=1

save.rd.length=1

save.d.ci.length=1

save.r.z.bca.length=1

save.r.z.ci.length=1

save.p=vector()

while(times<=simutime){

Y1=rnorm(vsample1[samplecount1],mean1,vsd1[sdcount1])

Y2=rnorm(vsample2[samplecount2],0,vsd2[sdcount2])

expd=vd[dcount]

if(expd==0){

expa=0.5

expeta=0

exprd=0

exprpb=0

}

if(expd==0.1){

expa=0.5282

expeta=0.0025

exprd=0.1

exprpb=0.0499

}

if(expd==0.2){

expa=0.56

expeta=0.0099

exprd=0.2

exprpb=0.0995

}

if(expd==0.5){

expa=0.64

expeta=0.0588

exprd=0.5

exprpb=0.2425

}

if(expd==0.8){

expa=0.714

expeta=0.1379

exprd=0.8

exprpb=0.3714

}

exp.r.z=(log(1+sqrt(expeta))-log(1-sqrt(expeta)))/2

dat=frame.boot(Y1,Y2)

asave=get.amethod(dat)

dsave=get.d(dat)

rdsave=get.rd(dat)

etasave=get.eta(dat)

rpbsave=get.rpb(dat)

r.zsave=get.r.z(dat)

poss.save=get.p(Y1,Y2)

a.bca.ci.save=A.bca.ci(Y1,Y2)

d.bca.ci.save=d.bca.ci(Y1,Y2)

rd.bca.ci.save=rd.bca.ci(Y1,Y2)

rpb.bca.ci.save=rpb.bca.ci(Y1,Y2)

r.z.bca.ci.save=r.z.bca.ci(Y1,Y2)

a.bca.min=a.bca.ci.save[1]

a.bca.max=a.bca.ci.save[2]

d.bca.min=d.bca.ci.save[1]

d.bca.max=d.bca.ci.save[2]

rd.bca.min=rd.bca.ci.save[1]

rd.bca.max=rd.bca.ci.save[2]

d.ci.min=ci.smd(smd=dsave,n.1=length(Y1),n.2=length(Y2))$Lower.Conf.Limit.smd

d.ci.max=ci.smd(smd=dsave,n.1=length(Y1),n.2=length(Y2))$Upper.Conf.Limit.smd

rpb.bca.min=rpb.bca.ci.save[1]

rpb.bca.max=rpb.bca.ci.save[2]

r.z.bca.min=r.z.bca.ci.save[1]

r.z.bca.max=r.z.bca.ci.save[2]

r.z.ci.min=r.zsave+((qnorm(0.025))/(sqrt(length(Y1)+length(Y2)-3)))

r.z.ci.max=r.zsave+((qnorm(0.975))/(sqrt(length(Y1)+length(Y2)-3)))

save.a=c(save.a,asave)

save.d=c(save.d,dsave)

save.rd=c(save.rd,rdsave)

save.eta=c(save.eta,etasave)

save.rpb=c(save.rpb,rpbsave)

save.r.z=c(save.r.z,r.zsave)

save.a.bca.min=c(save.a.bca.min,a.bca.min)

save.a.bca.max=c(save.a.bca.max,a.bca.max)

save.d.bca.min=c(save.d.bca.min,d.bca.min)

save.d.bca.max=c(save.d.bca.max,d.bca.max)

save.rd.bca.min=c(save.rd.bca.min,rd.bca.min)

save.rd.bca.max=c(save.rd.bca.max,rd.bca.max)

save.rpb.bca.min=c(save.rpb.bca.min,rpb.bca.min)

save.rpb.bca.max=c(save.rpb.bca.max,rpb.bca.max)

save.d.ci.min=c(save.d.ci.min,d.ci.min)

save.d.ci.max=c(save.d.ci.max,d.ci.max)

save.r.z.bca.min=c(save.r.z.bca.min,r.z.bca.min)

save.r.z.bca.max=c(save.r.z.bca.max,r.z.bca.max)

save.r.z.ci.min=c(save.r.z.ci.min,r.z.ci.min)

save.r.z.ci.max=c(save.r.z.ci.max,r.z.ci.max)

a.length=a.bca.max-a.bca.min

d.length=d.bca.max-d.bca.min

rd.length=rd.bca.max-rd.bca.min

rpb.length=rpb.bca.max-rpb.bca.min

d.ci.length=d.ci.max-d.ci.min

r.z.bca.length=r.z.bca.max-r.z.bca.min

r.z.ci.length=r.z.ci.max-r.z.ci.min

save.a.length=c(save.a.length,a.length)

save.d.length=c(save.d.length,d.length)

save.rd.length=c(save.rd.length,rd.length)

save.rpb.length=c(save.rpb.length,rpb.length)

save.d.ci.length=c(save.d.ci.length,d.ci.length)

save.r.z.bca.length=c(save.r.z.bca.length,r.z.bca.length)

save.r.z.ci.length=c(save.r.z.ci.length,r.z.ci.length)

save.p=c(save.p,poss.save)

print(c(times,condition))

times=times+1}

times=1

condition=condition+1

save.a=save.a[-1]

save.d=save.d[-1]

save.rd=save.rd[-1]

save.rpb=save.rpb[-1]

save.r.z=save.r.z[-1]

save.a.bca.min=save.a.bca.min[-1]

save.a.bca.max=save.a.bca.max[-1]

save.d.bca.min=save.d.bca.min[-1]

save.d.bca.max=save.d.bca.max[-1]

save.rd.bca.min=save.rd.bca.min[-1]

save.rd.bca.max=save.rd.bca.max[-1]

save.rpb.bca.min=save.rpb.bca.min[-1]

save.rpb.bca.max=save.rpb.bca.max[-1]

save.d.ci.min=save.d.ci.min[-1]

save.d.ci.max=save.d.ci.max[-1]

save.r.z.bca.min=save.r.z.bca.min[-1]

save.r.z.bca.max=save.r.z.bca.max[-1]

save.r.z.ci.min=save.r.z.ci.min[-1]

save.r.z.ci.max=save.r.z.ci.max[-1]

if(expd==0){

save.a.bca.min0=save.a.bca.min

save.a.bca.max0=save.a.bca.max

save.d.bca.min0=save.d.bca.min

save.d.bca.max0=save.d.bca.max

save.rd.bca.min0=save.rd.bca.min

save.rd.bca.max0=save.rd.bca.max

save.rpb.bca.min0=save.rpb.bca.min

save.rpb.bca.max0=save.rpb.bca.max

save.d.ci.min0=save.d.ci.min

save.d.ci.max0=save.d.ci.max

save.r.z.bca.min0=save.r.z.bca.min

save.r.z.bca.max0=save.r.z.bca.max

save.r.z.ci.min0=save.r.z.ci.min

save.r.z.ci.max0=save.r.z.ci.max

}

save.a.length=save.a.length[-1]

save.d.length=save.d.length[-1]

save.rd.length=save.rd.length[-1]

save.rpb.length=save.rpb.length[-1]

save.d.ci.length=save.d.ci.length[-1]

save.r.z.bca.length=save.r.z.bca.length[-1]

save.r.z.ci.length=save.r.z.ci.length[-1]

print("calculating cap")

a.cap=check.cap(save.a,save.a.bca.min,save.a.bca.max)/(simutime*simutime-simutime)

d.cap=check.cap(save.d,save.d.bca.min,save.d.bca.max)/(simutime*simutime-simutime)

rd.cap=check.cap(save.rd,save.rd.bca.min,save.rd.bca.max)/(simutime*simutime-simutime)

rpb.cap=check.cap(save.rpb,save.rpb.bca.min,save.rpb.bca.max)/(simutime*simutime-simutime)

ci.d.cap=check.cap(save.d,save.d.ci.min,save.d.ci.max)/(simutime*simutime-simutime)

r.z.bca.cap=check.cap(save.r.z,save.r.z.bca.min,save.r.z.bca.max)/(simutime*simutime-simutime)

r.z.ci.cap=check.cap(save.r.z,save.r.z.ci.min,save.r.z.ci.max)/(simutime*simutime-simutime)

#calculate the capture percentage of ES=!0 with ESCI=0.

a.cap0=check.cap(save.a,save.a.bca.min0,save.a.bca.max0)/(simutime*simutime-simutime)

d.cap0=check.cap(save.d,save.d.bca.min0,save.d.bca.max0)/(simutime*simutime-simutime)

rd.cap0=check.cap(save.rd,save.rd.bca.min0,save.rd.bca.max0)/(simutime*simutime-simutime)

rpb.cap0=check.cap(save.rpb,save.rpb.bca.min0,save.rpb.bca.max0)/(simutime*simutime-simutime)

ci.d.cap0=check.cap(save.d,save.d.ci.min0,save.d.ci.max0)/(simutime*simutime-simutime)

r.z.bca.cap0=check.cap(save.r.z,save.r.z.bca.min0,save.r.z.bca.max0)/(simutime*simutime-simutime)

r.z.ci.cap0=check.cap(save.r.z,save.r.z.ci.min0,save.r.z.ci.max0)/(simutime*simutime-simutime)

a.cov=check.cap(expa,save.a.bca.min,save.a.bca.max)/simutime

d.cov=check.cap(expd,save.d.bca.min,save.d.bca.max)/simutime

rd.cov=check.cap(exprd,save.rd.bca.min,save.rd.bca.max)/simutime

rpb.cov=check.cap(exprpb,save.rpb.bca.min,save.rpb.bca.max)/simutime

ci.d.cov=check.cap(expa,save.d.ci.min,save.d.ci.max)/simutime

ci.d.cov=check.cap(expa,save.d.ci.min,save.d.ci.max)/simutime

r.z.bca.cov=check.cap(exp.r.z,save.r.z.bca.min,save.r.z.bca.max)/simutime

r.z.ci.cov=check.cap(exp.r.z,save.r.z.ci.min,save.r.z.ci.max)/simutime

nhst.a=check.cap(0.5,save.a.bca.min,save.a.bca.max)

nhst.d=check.cap(0,save.d.bca.min,save.d.bca.max)

nhst.ci.d=check.cap(0,save.d.ci.min,save.d.ci.max)

nhst.rd=check.cap(0,save.rd.bca.min,save.rd.bca.max)

nhst.rpb=check.cap(0,save.rpb.bca.min,save.rpb.bca.max)

nhst.r.z.bca=check.cap(0,save.r.z.bca.min,save.r.z.bca.max)

nhst.r.z.ci=check.cap(0,save.r.z.ci.min,save.r.z.ci.max)

if(expd==0){

nhst.a=simutime-nhst.a

nhst.d=simutime-nhst.d

nhst.rd=simutime-nhst.rd

nhst.rpb=simutime-nhst.rpb

nhst.ci.d=simutime-nhst.ci.d

nhst.r.z.bca=simutime-nhst.r.z.bca

nhst.r.z.ci=simutime-nhst.r.z.ci

}

#for x.cap, x.cov the lower the better, a x.cap=1 means no ES in the orginal study#

#fall within the ES of replication study.

saveall=c(saveall,vsample1[samplecount1],vsample2[samplecount2],

vsd1[sdcount1],vsd2[sdcount2],vd[dcount],mean(save.p),

a.cap,d.cap,rd.cap,rpb.cap,ci.d.cap,r.z.bca.cap,r.z.ci.cap,

a.cap0,d.cap0,rd.cap0,rpb.cap0,ci.d.cap0,r.z.bca.cap0,r.z.ci.cap0,

a.cov,d.cov,rd.cov,rpb.cov,ci.d.cov,r.z.bca.cov,r.z.ci.cov,

nhst.a,nhst.d,nhst.rd,nhst.rpb,nhst.ci.d,nhst.r.z.bca,nhst.r.z.ci,

mean(save.a.length),mean(save.d.length),

mean(save.rd.length),mean(save.rpb.length),

mean(save.d.ci.length),mean(save.r.z.bca.length),mean(save.r.z.ci.length))

dcount=dcount+1}

dcount=1

sdcount2=sdcount2+1}

sdcount2=1

sdcount1=sdcount1+1}

sdcount1=1

samplecount2=samplecount2+1}

samplecount2=1

samplecount1=samplecount1+1}

saveall=saveall[-1]

savepp=matrix(saveall,41,)

write.table(savepp,file="c:\\data1NS.csv",sep=",")

**Study 2**

install.packages("boot", dependencies = TRUE)

install.packages("lsr", dependencies = TRUE)

install.packages("doParallel", dependencies = TRUE)

install.packages("MBESS", dependencies = TRUE)

install.packages("psych", dependencies = TRUE)

library(MBESS)

library(boot)

library(lsr)

library(psych)

library(doParallel)

clus.h=4

boottime=1000

simutime=1000

cll <- makeCluster(clus.h)

registerDoParallel(cll)

set.seed(201701)

get.p=function(a,b){

sig.test=t.test(a,b,paired = FALSE,var.equal = FALSE)$p.value

if(sig.test<0.05){sig.use=1}

if(sig.test>=0.05){sig.use=0}

return(sig.use)

}

get.amethod=function(dat){

Y1 <- dat[which(dat[,1] ==1, arr.ind = T),2]

Y2 <- dat[which(dat[,1] ==2, arr.ind = T),2]

part1=length(Y1)

part2=length(Y2)

countA=0

ut1=1

while(ut1<=part1){

ut2=1

while(ut2<=part2){

if(Y1[ut1]>Y2[ut2]){

countA=countA+1

}

if(Y1[ut1]==Y2[ut2]){

countA=countA+0.5

}

ut2=ut2+1

}

ut1=ut1+1

}

amethod=countA/(part1*part2)

return(amethod)

}

get.d=function(dat){

likeY1 <- dat[which(dat[,1] ==1, arr.ind = T),2]

likeY2 <- dat[which(dat[,1] ==2, arr.ind = T),2]

p1=(length(likeY1))/(length(likeY1)+length(likeY2))

p2=(length(likeY2))/(length(likeY1)+length(likeY2))

cohend=(mean(likeY1)-mean(likeY2))/((p1*var(likeY1)+p2*var(likeY2))^0.5)

return(cohend)

}

get.rd=function(dat){

likeY1 <- dat[which(dat[,1] ==1, arr.ind = T),2]

likeY2 <- dat[which(dat[,1] ==2, arr.ind = T),2]

part1=length(likeY1)

part2=length(likeY2)

robustv=(winsor.var(likeY1,trim=0.2)*(part1-1)+winsor.var(likeY2,trim=0.2)*(part2-1))/(part1+part2-2)

cohendr=0.642*((mean(likeY1,trim=0.2)-mean(likeY2,trim=0.2))/((robustv)^0.5))

return(cohendr)

}

get.rpb=function(dat){

likeY1 <- dat[which(dat[,1] ==1, arr.ind = T),2]

likeY2 <- dat[which(dat[,1] ==2, arr.ind = T),2]

p1=(length(likeY1))/(length(likeY1)+length(likeY2))

p2=(length(likeY2))/(length(likeY1)+length(likeY2))

rpb=(mean(likeY1)-mean(likeY2))/((p1*var(likeY1)+p2*var(likeY2))/(p1*p2)+(mean(likeY1)-mean(likeY2))^2)^0.5

return(rpb)}

get.eta=function(dat){

likeY1 <- dat[which(dat[,1] ==1, arr.ind = T),2]

likeY2 <- dat[which(dat[,1] ==2, arr.ind = T),2]

likeall=c(likeY1,likeY2)

ssbetween=(mean(likeall)-mean(likeY1))^2*length(likeY1)+(mean(likeall)-mean(likeY2))^2*length(likeY2)

sstotal=sum((likeY1-mean(likeall))^2)+sum((likeY2-mean(likeall))^2)

eta=ssbetween/sstotal

return(eta)}

get.r.z=function(dat){

cor.u=cor(dat[,1],dat[,2])

z.u=(log(1+cor.u)-log(1-cor.u))/2

return(z.u)

}

frame.boot=function(Y1,Y2){

n1 <- length(Y1)

n2 <- length(Y2)

n <- n1+n2

dat <- array(0, dim=c(n,2))

dat[1:n1,1] <- 1

dat[(n1+1):n,1] <- 2

dat[1:n1,2] <- Y1

dat[(n1+1):n,2] <- Y2

return(dat)

}

f.a <- function(d, i){

d2 <- d[i,]

return(get.amethod(d2))

}

f.d <- function(d, i){

d2 <- d[i,]

return(get.d(d2))

}

f.rd <- function(d, i){

d2 <- d[i,]

return(get.rd(d2))

}

f.rpb <- function(d, i){

d2 <- d[i,]

return(get.rpb(d2))

}

f.eta <- function(d, i){

d2 <- d[i,]

return(get.eta(d2))

}

f.r.z <- function(d, i){

d2 <- d[i,]

return(get.r.z(d2))

}

A.bca.ci=function(Y1,Y2){

dat=frame.boot(Y1,Y2)

boot.data <- boot(dat, f.a, R=boottime, parallel = c("snow"), cl=clus.h)

save.all=boot.ci(boot.data, type = c("bca"))

save.min=save.all$"bca"[4]

save.max=save.all$"bca"[5]

return(c(save.min,save.max))

}

d.bca.ci=function(Y1,Y2){

dat=frame.boot(Y1,Y2)

boot.data <- boot(dat, f.d, R=boottime, parallel = c("snow"), cl=clus.h)

save.all=boot.ci(boot.data, type = c("bca"))

save.min=save.all$"bca"[4]

save.max=save.all$"bca"[5]

return(c(save.min,save.max))

}

rd.bca.ci=function(Y1,Y2){

dat=frame.boot(Y1,Y2)

boot.data <- boot(dat, f.rd, R=boottime, parallel = c("snow"), cl=clus.h)

save.all=boot.ci(boot.data, type = c("bca"))

save.min=save.all$"bca"[4]

save.max=save.all$"bca"[5]

return(c(save.min,save.max))

}

rpb.bca.ci=function(Y1,Y2){

dat=frame.boot(Y1,Y2)

boot.data <- boot(dat, f.rpb, R=boottime, parallel = c("snow"), cl=clus.h)

save.all=boot.ci(boot.data, type = c("bca"))

save.min=save.all$"bca"[4]

save.max=save.all$"bca"[5]

return(c(save.min,save.max))

}

eta.bca.ci=function(Y1,Y2){

dat=frame.boot(Y1,Y2)

boot.data <- boot(dat, f.eta, R=boottime, parallel = c("snow"), cl=clus.h)

save.all=boot.ci(boot.data, type = c("bca"))

save.min=save.all$"bca"[4]

save.max=save.all$"bca"[5]

return(c(save.min,save.max))

}

r.z.bca.ci=function(Y1,Y2){

dat=frame.boot(Y1,Y2)

boot.data <- boot(dat, f.r.z, R=boottime, parallel = c("snow"), cl=clus.h)

save.all=boot.ci(boot.data, type = c("bca"))

save.min=save.all$"bca"[4]

save.max=save.all$"bca"[5]

return(c(save.min,save.max))

}

check.include=function(test,min,max){

ans=0

if(test>max){ans=1}

if(test<min){ans=1}

return(ans)}

check.cap=function(resv,minv,maxv){

ans.u=0

resv.n=1

while(resv.n<=length(resv)){

resu=resv[resv.n]

min.v=1

while(min.v<=length(minv)){

minu=minv[min.v]

maxu=maxv[min.v]

ans.u=ans.u+check.include(resu,minu,maxu)

min.v=min.v+1}

resv.n=resv.n+1}

return(ans.u)}

######################

vsample1=c(100,50,25)

vsample2=c(1)

vsd1=c(0.5,1,4)

vsd2=c(1)

vd=c(0,0.1,0.2,0.5,0.8)

samplecount1=1

samplecount2=1

sdcount1=1

sdcount2=1

dcount=1

times=1

saveall=1

condition=1

while(samplecount2<=length(vsample2)){

while(sdcount1<=length(vsd1)){

while(sdcount2<=length(vsd2)){

while(dcount<=length(vd)){

while(samplecount1<=length(vsample1)){

vsample2=vsample1[samplecount1]

p1=(vsample1[samplecount1])/(vsample1[samplecount1]+vsample2[samplecount2])

p2=(vsample2[samplecount2])/(vsample1[samplecount1]+vsample2[samplecount2])

pooledsd=(vsd1[sdcount1]^2*p1+vsd2[sdcount2]^2*p2)^0.5

mean1=(vd[dcount])*pooledsd

save.a=1

save.d=1

save.rd=1

save.rpb=1

save.eta=1

save.r.z=1

save.a.bca.min=1

save.a.bca.max=1

save.d.bca.min=1

save.d.bca.max=1

save.rd.bca.min=1

save.rd.bca.max=1

save.rpb.bca.min=1

save.rpb.bca.max=1

save.d.ci.min=1

save.d.ci.max=1

save.r.z.bca.min=1

save.r.z.bca.max=1

save.r.z.ci.min=1

save.r.z.ci.max=1

save.a.length=1

save.d.length=1

save.rpb.length=1

save.rd.length=1

save.d.ci.length=1

save.r.z.bca.length=1

save.r.z.ci.length=1

save.p=vector()

while(times<=simutime){

Y1=rnorm(vsample1[samplecount1],mean1,vsd1[sdcount1])

Y2=rnorm(vsample2[samplecount2],0,vsd2[sdcount2])

expd=vd[dcount]

if(expd==0){

expa=0.5

expeta=0

exprd=0

exprpb=0

}

if(expd==0.1){

expa=0.5282

expeta=0.0025

exprd=0.1

exprpb=0.0499

}

if(expd==0.2){

expa=0.56

expeta=0.0099

exprd=0.2

exprpb=0.0995

}

if(expd==0.5){

expa=0.64

expeta=0.0588

exprd=0.5

exprpb=0.2425

}

if(expd==0.8){

expa=0.714

expeta=0.1379

exprd=0.8

exprpb=0.3714

}

exp.r.z=(log(1+sqrt(expeta))-log(1-sqrt(expeta)))/2

dat=frame.boot(Y1,Y2)

asave=get.amethod(dat)

dsave=get.d(dat)

rdsave=get.rd(dat)

etasave=get.eta(dat)

rpbsave=get.rpb(dat)

r.zsave=get.r.z(dat)

poss.save=get.p(Y1,Y2)

a.bca.ci.save=A.bca.ci(Y1,Y2)

d.bca.ci.save=d.bca.ci(Y1,Y2)

rd.bca.ci.save=rd.bca.ci(Y1,Y2)

rpb.bca.ci.save=rpb.bca.ci(Y1,Y2)

r.z.bca.ci.save=r.z.bca.ci(Y1,Y2)

a.bca.min=a.bca.ci.save[1]

a.bca.max=a.bca.ci.save[2]

d.bca.min=d.bca.ci.save[1]

d.bca.max=d.bca.ci.save[2]

rd.bca.min=rd.bca.ci.save[1]

rd.bca.max=rd.bca.ci.save[2]

d.ci.min=ci.smd(smd=dsave,n.1=length(Y1),n.2=length(Y2))$Lower.Conf.Limit.smd

d.ci.max=ci.smd(smd=dsave,n.1=length(Y1),n.2=length(Y2))$Upper.Conf.Limit.smd

rpb.bca.min=rpb.bca.ci.save[1]

rpb.bca.max=rpb.bca.ci.save[2]

r.z.bca.min=r.z.bca.ci.save[1]

r.z.bca.max=r.z.bca.ci.save[2]

r.z.ci.min=r.zsave+((qnorm(0.025))/(sqrt(length(Y1)+length(Y2)-3)))

r.z.ci.max=r.zsave+((qnorm(0.975))/(sqrt(length(Y1)+length(Y2)-3)))

save.a=c(save.a,asave)

save.d=c(save.d,dsave)

save.rd=c(save.rd,rdsave)

save.eta=c(save.eta,etasave)

save.rpb=c(save.rpb,rpbsave)

save.r.z=c(save.r.z,r.zsave)

save.a.bca.min=c(save.a.bca.min,a.bca.min)

save.a.bca.max=c(save.a.bca.max,a.bca.max)

save.d.bca.min=c(save.d.bca.min,d.bca.min)

save.d.bca.max=c(save.d.bca.max,d.bca.max)

save.rd.bca.min=c(save.rd.bca.min,rd.bca.min)

save.rd.bca.max=c(save.rd.bca.max,rd.bca.max)

save.rpb.bca.min=c(save.rpb.bca.min,rpb.bca.min)

save.rpb.bca.max=c(save.rpb.bca.max,rpb.bca.max)

save.d.ci.min=c(save.d.ci.min,d.ci.min)

save.d.ci.max=c(save.d.ci.max,d.ci.max)

save.r.z.bca.min=c(save.r.z.bca.min,r.z.bca.min)

save.r.z.bca.max=c(save.r.z.bca.max,r.z.bca.max)

save.r.z.ci.min=c(save.r.z.ci.min,r.z.ci.min)

save.r.z.ci.max=c(save.r.z.ci.max,r.z.ci.max)

a.length=a.bca.max-a.bca.min

d.length=d.bca.max-d.bca.min

rd.length=rd.bca.max-rd.bca.min

rpb.length=rpb.bca.max-rpb.bca.min

d.ci.length=d.ci.max-d.ci.min

r.z.bca.length=r.z.bca.max-r.z.bca.min

r.z.ci.length=r.z.ci.max-r.z.ci.min

save.a.length=c(save.a.length,a.length)

save.d.length=c(save.d.length,d.length)

save.rd.length=c(save.rd.length,rd.length)

save.rpb.length=c(save.rpb.length,rpb.length)

save.d.ci.length=c(save.d.ci.length,d.ci.length)

save.r.z.bca.length=c(save.r.z.bca.length,r.z.bca.length)

save.r.z.ci.length=c(save.r.z.ci.length,r.z.ci.length)

save.p=c(save.p,poss.save)

print(c(times,condition))

times=times+1}

times=1

condition=condition+1

save.a=save.a[-1]

save.d=save.d[-1]

save.rd=save.rd[-1]

save.rpb=save.rpb[-1]

save.r.z=save.r.z[-1]

save.a.bca.min=save.a.bca.min[-1]

save.a.bca.max=save.a.bca.max[-1]

save.d.bca.min=save.d.bca.min[-1]

save.d.bca.max=save.d.bca.max[-1]

save.rd.bca.min=save.rd.bca.min[-1]

save.rd.bca.max=save.rd.bca.max[-1]

save.rpb.bca.min=save.rpb.bca.min[-1]

save.rpb.bca.max=save.rpb.bca.max[-1]

save.d.ci.min=save.d.ci.min[-1]

save.d.ci.max=save.d.ci.max[-1]

save.r.z.bca.min=save.r.z.bca.min[-1]

save.r.z.bca.max=save.r.z.bca.max[-1]

save.r.z.ci.min=save.r.z.ci.min[-1]

save.r.z.ci.max=save.r.z.ci.max[-1]

if(vsample1[samplecount1]==100){

save.a.bca.min0=save.a.bca.min

save.a.bca.max0=save.a.bca.max

save.d.bca.min0=save.d.bca.min

save.d.bca.max0=save.d.bca.max

save.rd.bca.min0=save.rd.bca.min

save.rd.bca.max0=save.rd.bca.max

save.rpb.bca.min0=save.rpb.bca.min

save.rpb.bca.max0=save.rpb.bca.max

save.d.ci.min0=save.d.ci.min

save.d.ci.max0=save.d.ci.max

save.r.z.bca.min0=save.r.z.bca.min

save.r.z.bca.max0=save.r.z.bca.max

save.r.z.ci.min0=save.r.z.ci.min

save.r.z.ci.max0=save.r.z.ci.max

}

save.a.length=save.a.length[-1]

save.d.length=save.d.length[-1]

save.rd.length=save.rd.length[-1]

save.rpb.length=save.rpb.length[-1]

save.d.ci.length=save.d.ci.length[-1]

save.r.z.bca.length=save.r.z.bca.length[-1]

save.r.z.ci.length=save.r.z.ci.length[-1]

print("calculating cap")

a.cap=check.cap(save.a,save.a.bca.min,save.a.bca.max)/(simutime*simutime-simutime)

d.cap=check.cap(save.d,save.d.bca.min,save.d.bca.max)/(simutime*simutime-simutime)

rd.cap=check.cap(save.rd,save.rd.bca.min,save.rd.bca.max)/(simutime*simutime-simutime)

rpb.cap=check.cap(save.rpb,save.rpb.bca.min,save.rpb.bca.max)/(simutime*simutime-simutime)

ci.d.cap=check.cap(save.d,save.d.ci.min,save.d.ci.max)/(simutime*simutime-simutime)

r.z.bca.cap=check.cap(save.r.z,save.r.z.bca.min,save.r.z.bca.max)/(simutime*simutime-simutime)

r.z.ci.cap=check.cap(save.r.z,save.r.z.ci.min,save.r.z.ci.max)/(simutime*simutime-simutime)

#calculate the capture percentage of when the sample size of ES is 25,50,100

#and sample size of ESCI is 100.

a.cap0=check.cap(save.a,save.a.bca.min0,save.a.bca.max0)/(simutime*simutime-simutime)

d.cap0=check.cap(save.d,save.d.bca.min0,save.d.bca.max0)/(simutime*simutime-simutime)

rd.cap0=check.cap(save.rd,save.rd.bca.min0,save.rd.bca.max0)/(simutime*simutime-simutime)

rpb.cap0=check.cap(save.rpb,save.rpb.bca.min0,save.rpb.bca.max0)/(simutime*simutime-simutime)

ci.d.cap0=check.cap(save.d,save.d.ci.min0,save.d.ci.max0)/(simutime*simutime-simutime)

r.z.bca.cap0=check.cap(save.r.z,save.r.z.bca.min0,save.r.z.bca.max0)/(simutime*simutime-simutime)

r.z.ci.cap0=check.cap(save.r.z,save.r.z.ci.min0,save.r.z.ci.max0)/(simutime*simutime-simutime)

a.cov=check.cap(expa,save.a.bca.min,save.a.bca.max)/simutime

d.cov=check.cap(expd,save.d.bca.min,save.d.bca.max)/simutime

rd.cov=check.cap(exprd,save.rd.bca.min,save.rd.bca.max)/simutime

rpb.cov=check.cap(exprpb,save.rpb.bca.min,save.rpb.bca.max)/simutime

ci.d.cov=check.cap(expa,save.d.ci.min,save.d.ci.max)/simutime

ci.d.cov=check.cap(expa,save.d.ci.min,save.d.ci.max)/simutime

r.z.bca.cov=check.cap(exp.r.z,save.r.z.bca.min,save.r.z.bca.max)/simutime

r.z.ci.cov=check.cap(exp.r.z,save.r.z.ci.min,save.r.z.ci.max)/simutime

nhst.a=check.cap(0.5,save.a.bca.min,save.a.bca.max)

nhst.d=check.cap(0,save.d.bca.min,save.d.bca.max)

nhst.ci.d=check.cap(0,save.d.ci.min,save.d.ci.max)

nhst.rd=check.cap(0,save.rd.bca.min,save.rd.bca.max)

nhst.rpb=check.cap(0,save.rpb.bca.min,save.rpb.bca.max)

nhst.r.z.bca=check.cap(0,save.r.z.bca.min,save.r.z.bca.max)

nhst.r.z.ci=check.cap(0,save.r.z.ci.min,save.r.z.ci.max)

if(expd==0){

nhst.a=simutime-nhst.a

nhst.d=simutime-nhst.d

nhst.rd=simutime-nhst.rd

nhst.rpb=simutime-nhst.rpb

nhst.ci.d=simutime-nhst.ci.d

nhst.r.z.bca=simutime-nhst.r.z.bca

nhst.r.z.ci=simutime-nhst.r.z.ci

}

#for x.cap, x.cov the lower the better#

saveall=c(saveall,vsample1[samplecount1],vsample2[samplecount2],

vsd1[sdcount1],vsd2[sdcount2],vd[dcount],mean(save.p),

a.cap,d.cap,rd.cap,rpb.cap,ci.d.cap,r.z.bca.cap,r.z.ci.cap,

a.cap0,d.cap0,rd.cap0,rpb.cap0,ci.d.cap0,r.z.bca.cap0,r.z.ci.cap0,

a.cov,d.cov,rd.cov,rpb.cov,ci.d.cov,r.z.bca.cov,r.z.ci.cov,

nhst.a,nhst.d,nhst.rd,nhst.rpb,nhst.ci.d,nhst.r.z.bca,nhst.r.z.ci,

mean(save.a.length),mean(save.d.length),

mean(save.rd.length),mean(save.rpb.length),

mean(save.d.ci.length),mean(save.r.z.bca.length),mean(save.r.z.ci.length))

samplecount1=samplecount1+1}

samplecount1=1

dcount=dcount+1}

dcount=1

sdcount2=sdcount2+1}

sdcount2=1

sdcount1=sdcount1+1}

sdcount1=1

samplecount2=samplecount2+1}

saveall=saveall[-1]

savepp=matrix(saveall,41,)

write.table(savepp,file="c:\\data1TS.csv",sep=",")
